# Rare renal tumor: a case report of collecting duct carcinoma and literature review

**DOI:** 10.3389/fonc.2026.1674239

**Published:** 2026-03-05

**Authors:** Cheng Zhu, Yingfang Zhang, Zhong Tian, Tingting Yang, Guang Han, Huangyu Luo, Bo Yu, Neng Zhang, Ni Fu

**Affiliations:** 1 Department of Urology, The Second Affiliated Hospital of Zunyi Medical University, Zunyi, China; 2 Department of Nursing, The Affiliated Hospital of Zunyi Medical University, Zunyi, China; 3 Department of Pathology, The Affiliated Hospital of Zunyi Medical University, Zunyi, China; 4 Department of Urology, The Affiliated Hospital of Zunyi Medical University, Zunyi, China

**Keywords:** aggressive renal cell carcinoma, case report, collecting duct carcinoma, differential diagnosis, metastasis, rare disease

## Abstract

Collecting duct carcinoma (CDC), also known as Bellini duct carcinoma, is an extremely rare and highly aggressive subtype of renal cell carcinoma. Most patients present with metastasis at the time of diagnosis, and the prognosis for CDC patients is generally poor. Surgery is the primary treatment approach. In this article, we report a case of CDC presenting primarily with hematuria and provide a comprehensive review of the published literature concerning its diagnosis, treatment, and prognosis. The aim is to enhance understanding of this rare renal malignancy by elucidating its clinical and pathological characteristics and supplementing relevant treatment experiences.

## Introduction

Renal cell carcinoma (RCC) is recognized as one of the most lethal malignancies, accounting for approximately 3% to 5% of all adult cancers (1). RCC comprises numerous subtypes. Collecting duct carcinoma (CDC), also known as Bellini duct carcinoma, is an extremely rare subtype of RCC, representing less than 2% of all cases (2–4). It is a malignant tumor originating from the collecting duct system in the renal medulla. CDC is classified as a high-grade renal cell carcinoma, characterized by high aggressiveness and generally poor prognosis. The majority of patients present with metastasis at the time of diagnosis. Across different studies, the age of onset ranges from 16 to 90 years, with a median age between 53 and 66 years (5, 6).

The origin of CDC was first described by Cromie et al. in 1979 (7). It was identified as a distinct subtype of renal cell carcinoma by Fleming et al. in 1986 (8), and was first categorized as a subclass of RCC in the 1997 Heidelberg-Rochester classification by Kovacs et al. (9). The World Health Organization (WHO) classified CDC as a specific type of RCC in 2004 (10).

Due to its rarity, aggressiveness, and unfavorable prognosis, CDC poses significant challenges in clinical urological practice. In this article, we report a case of CDC presenting primarily with hematuria and provide a review of the relevant published literature (**Table 1**). The aim is to enhance the understanding of this rare renal malignancy by elucidating its clinical and pathological characteristics and supplementing relevant treatment experience.

**Table 1:** Summary of CDC published in the past decade. (This series includes surgically treated cases that were non-metastatic at presentation).

Case no	First author, year	Age (years) /sex /side	Size(cm)	Symptoms	Metastatic site(s)	Surgery	Adjuvant therapy	Follow -up (months)
1	Yin et al., 2016	51/M/L	3.5	Hematuria	LN	RN	Everolimus	9
2	Rimar et al., 2016	53/F/L	4.6×3.1×1.6	Hematuria	Lung Bone LN	No	Nivolumab	3
3	(11)	54/M/L	17×14×19	Left-sided chest pain, Fever	Heart Lung LN	RN	Pazopanib	4
4	(12)	54/M/R	NR	Swelling of the left jaw	Oral cavity	NR	NR	NR
5	Klang et al., 2017	32/M/R	NR	Fever Back pain Leg pain	Lung Bone Liver Spleen	No	Gemcitabine and Carboplatin	NR
6	Yasuoka et al., 2018	73/M/R	5.5	Fever Back pain	Lung Liver Adrenal gland LN	RN	Nivolumab	NR
7	Norbert et al., 2019	31/F/R	3.1×3.9	Back pain Flank pain	Liver Bone LN	PN	NR	NR
8	Gong et al., 2019	53/M/B	L: 9.4 R: 8.1	Flank pain	LN Lung Bone	NR	No	7
9	Mathisekara et al., 2019	43/F/L	5.5×2.5	Fever Abdominal pain	Liver Lung	RN+LND	NR	1
10	(13)	52/M/L	5.2×6.4	Weight loss Fatigue Skin nodules	Liver Lung LN Skin	No	Gemcitabine and Cisplatin	4
11	Khoury et al., 2020	41/M/L	5	Flank pain Hematuria	LN Psoas major	RN	Gemcitabine and Cisplatin	24
12	(14)	73/M/R	4.2×3.7	NR	Bone	No	Neoantigen immunotherapy	9
13	He et al., 2020	68/M/L	NR	Low fever Chest tightness Back swelling	LN Liver	RN	NR	1
14	Fulop et al., 2020	42/M/R	12.6× 12.1× 14.6	Right-sided chest pain	No	RN	NR	21
15	Hasan et al., 2020	45/M/R	6×3	Cough Dyspnea Weight loss	Lungs Bones Adrenal gland	No	NR	6
16	Houmaidi et al., 2020	68/F/L	12×9×11	Flank pain	LN	RN	Gemcitabine and Cisplatin	48
17	Matej et al., 2020	61/F/R	4.0×3.5	Flank pain Hematuria	Liver Bone Lung LN	RN	MVAC and Sorafenib	18
18	Zhou et al., 2021	67/M/L	5.2×4.3	Flank pain	Lung Bone	RN	Axitinib and Sintilimab	11
19	Wu et al., 2021	59/F/L	5.0	Back pain	LN Lung Adrenal gland	RN	Sorafenib and Nivolumab	78
20	(15)	65/M/L	NR	Fever Lower Extremity pain	Subcutaneous tissue Muscle	NR	NR	NR
21	Fuu et al., 2022	70/M/R	NR	No	Lung Liver	RN	Nivolumab and Ipilimumab	36
22	Tamada et al., 2022	62/M/R 71/M/L	2.5 7.0	Loss of appetite Back pain	Bone Adrenal gland Lung	No	Pembrolizumab and Axitinib	7 9
23	(15)	60/M/R	6.6×6.1	Cough Back Pain Weight loss	Lung Liver LN	No	Carrelizumab and Sorafenib	2
24	Deng et al., 2023	52/F/R	7.1×6.4×7.6	No	Lung Liver Bone	No	NR	NR
25	Colef et al., 2024	69/M/L	NR	Flank pain Swelling of lower extremities	NR	RN	NR	NR
26	Satoki et al., 2024	67/F/L	9.0×7.0	NR	Liver	RN	Pembrolizumab	60
27	Gentry et al., 2024	22/F/R	2.4×2.0×2.1	Flank pain	No	RARN+LND	No	NR
28	Rahman et al., 2024	71/M/L	8×6	Flank pain Hematuria Fever	LN Bone	RN	Gemcitabine and Carboplatin	2
29	Wang et al., 2024	73/M/R	3.5×2.2×2.0	Flank Pain	Bone	RN	NR	NR
30	Sanjakanth et al., 2024	64/M/R	6.2×5.2	Flank pain Hematuria	NR	RN	NR	NR
31	Assis et al., 2024	36/F/L	NR	Post-kidney transplant	Lung LN Ovarie Fallopian tube	RN	Gemcitabine and Cisplatin	72
32	Franco et al., 2024	54/M/L	4	Flank pain	Bone LN	No	Pembrolizumab	6
33	Luo et al., 2025	49/M/L	NR	Flank pain Hematuria	LN Liver Pleura	RN	NR	4

M, male; F, female; L, left; R, right; B, Bilateral; NR, No Reported; CDC, Collecting duct carcinoma; UC, Urothelial Carcinoma; pRCC, papillary renal cell carcinoma; LN, Lymph nodes; RN, Radical nephrectomy; RARN, Robot-assisted radical nephrectomy; LND, Lymph node dissection; PN, Partial nnephrectomy; No, The patient was not surgically treated.

## Case report

**Figure 1:**
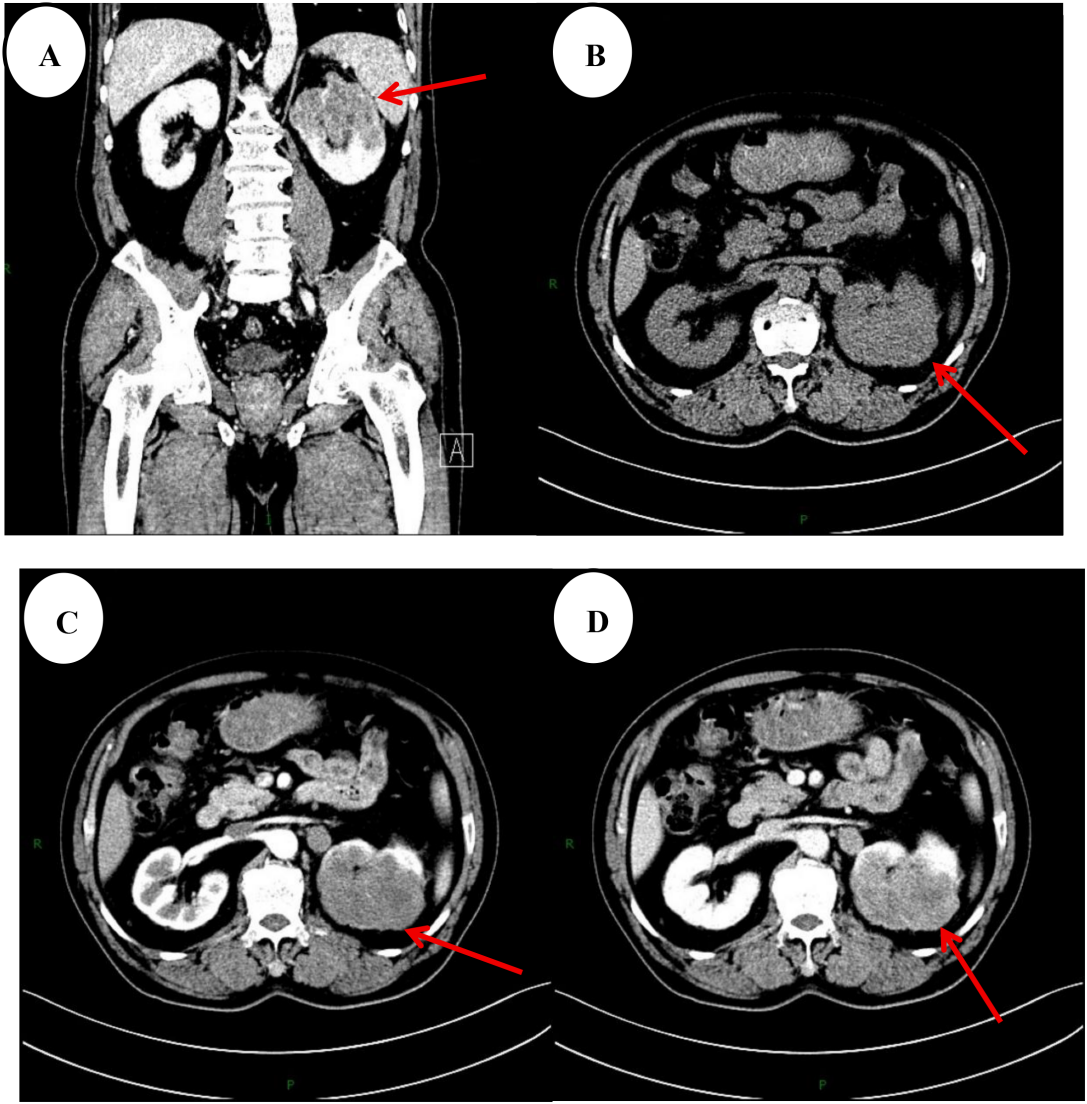
**(A–D)** Unenhanced CT images demonstrate a soft-tissue mass at the upper pole of the left kidney with ill-defined margins and heterogeneous attenuation. Contrast-enhanced imaging reveals mild, heterogeneous enhancement within the mass.

**Figure 2:**
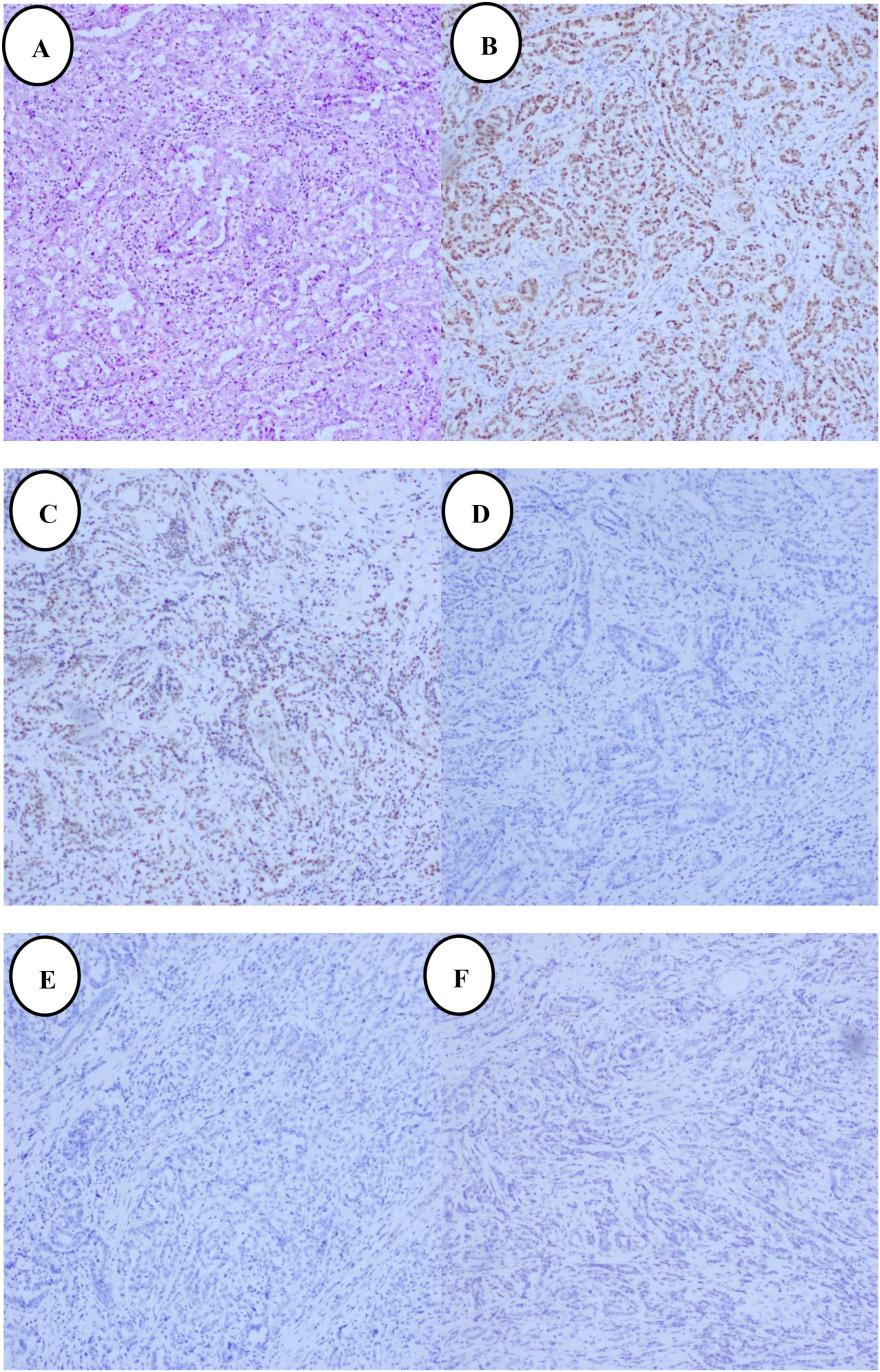
**(A)** H&E, 10×: Densely packed neoplastic cells forming irregular sheets amid desmoplastic stroma. **(B)** PAX-8 immunohistochemistry: Neoplastic cells show nuclear positivity. **(C)** INI-1 immunohistochemistry: Neoplastic cells show nuclear positivity. **(D–F)** Immunohistochemical analysis demonstrated negative staining for OCT3/4, GATA3, and p63 in tumor cells.

A 66-year-old male was admitted to the hospital due to a one-week history of hematuria accompanied by pain during urination without any obvious cause. He reported no abdominal pain or distension, no cough or sputum production, no fever, chills, fatigue, night sweats, or other discomforts. His mental state and appetite were good, with normal bowel and urinary habits, and no significant weight loss or gain was observed recently. The patient had a previous history of good health, denying any chronic diseases (such as hypertension, diabetes, chronic kidney disease, etc.), any history of infectious diseases, and any allergies to medications or food. He also reported no family history of hereditary disorders. The patient’s overall physical condition and performance status were good. Urinalysis indicated a red blood cell count of 1298/µL and hematuria (3+). A complete blood count showed a hemoglobin level of 107 g/L, suggesting mild anemia. The remaining blood tests revealed no significant abnormalities. Regarding imaging studies, a renal ultrasound demonstrated a hypoechoic mass measuring approximately 58×50 mm at the upper pole of the left kidney. Subsequent non-contrast and contrast-enhanced computed tomography (CT) scans revealed the following findings (**Figure 1**): a soft-tissue mass at the upper pole of the left kidney, measuring approximately 75×59×67 mm, with ill-defined borders and heterogeneous density. The lesion showed mild, heterogeneous enhancement on contrast administration, raising suspicion for a malignant neoplasm. The left adrenal gland appeared thickened with heterogeneous enhancement, suggestive of possible metastatic involvement. Additionally, there were multiple and partially enlarged retroperitoneal lymph nodes. Following completion of the preoperative evaluation and exclusion of surgical contraindications, the patient underwent a laparoscopic radical left nephrectomy. During the procedure, the perirenal fascia was incised at the lower pole of the left kidney. The proximal ureter was bluntly dissected free up to the renal pelvis, where enlarged hilar lymphatic vessels and adhesions in the surrounding tissues were observed. After blunt dissection and isolation of the left renal artery and vein, these vessels along with the ureter were ligated and divided. The superior edge of the left perirenal fascia was then incised and stripped, exposing the upper pole of the left kidney and the adrenal region. The left kidney was completely resected *en bloc* outside Gerota’s fascia. The cut surface of the tumor area appeared grayish-white and grayish-yellow in color, with a solid consistency. Macroscopic examination revealed invasion of the renal capsule. Postoperative pathological examination (**Figure 2**) identified a neoplastic lesion in the left kidney. The tumor invaded into the perirenal and renal sinus fat. The surgical margins of the ureter and blood vessels were free of tumor involvement. Combined with immunohistochemical staining results: PAX-8 (+), GATA3 (-), P63 (-), OCT3/4 (-), INI1 (+), PAX-2 (scattered +), EMA (partial +), Vimentin (+), CK-L (+), CK-H (-), CK7 (-), CD10 (-), CD117 (-), CEA (-), CA9 (-), Villin (-), and ki-67 (approximately 60% positive in hotspot areas). Based on the pathological findings, the patient was ultimately diagnosed with collecting duct carcinoma (CDC) of the left kidney. As of the last follow-up at 13 months postoperatively, there has been no evidence of tumor recurrence or metastasis, and the patient remains alive. Given that preoperative assessment indicated possible tumor metastasis and that systemic therapy would be required, the patient and their family were informed of this situation. They expressed that, due to financial reasons, they would not proceed with systemic therapy at this time.

## Discussion

Collecting duct carcinoma (CDC) is one of the most uncommon types of kidney cancer. The vast majority of patients present with advanced disease and metastasis at the time of diagnosis (16). **Table 2** summarizes evidence of lymph node involvement and distant metastasis in CDC from previously published literature. The median survival for CDC ranges from 10 to 13 months (5, 6, 22), and most patients die within two years of diagnosis, indicating a very poor prognosis. In the present case, the patient has shown no signs of recurrence during the 13-month follow-up period since surgery and remains alive. In previous research, the first large-scale clinical dataset describing CDC was collected in 2006 by Tokuda et al. (5) from a multicenter study across 281 institutions in Japan, involving 81 cases of CDC with metastasis. Among these, 43 patients underwent lymph node dissection, with 19 (44.2%) found to have regional lymph node metastasis. Furthermore, 26 patients (32.1%) presented with distant metastasis at the onset of the disease, with lung metastasis (14 cases) being the most common, followed by bone metastasis (13 cases). The median follow-up time was 15 months, and the 1-year, 3-year, 5-year, and 10-year disease-specific survival rates were 69.0%, 45.3%, 34.3%, and 13.7%, respectively. In 2007, Karakiewicz et al. (6) identified 5246 cases of clear cell renal cell carcinoma and 41 cases (0.6%) of CDC from a cohort of 6608 patients who underwent kidney cancer surgery. Lymph node metastasis was present in 48.8% of CDC cases, and distant metastasis in 19.5%. Notably, 76% of CDC patients were staged as pT3 post-surgery, with the majority having Fuhrman grades III (56%) and IV (22%). In contrast, only 37% of clear cell renal carcinoma cases were pT3. In 2017, Sui et al. (17) identified 577 CDC patients using the National Cancer Database (NCDB). Among them, 70.7% presented with metastatic disease at diagnosis. Distant metastasis was observed in 184 cases, with lung involvement accounting for 57%, and lymph node metastasis accounted for 48%. In 2022, Xie et al. (18) retrospectively collected data from approximately 8500 patients who underwent nephrectomy at the Mayo Clinic between 1970 and 2018. They identified 21 patients with pathologically confirmed CDC. Among these, 11 (52%) had lymph node involvement, and 7 (33%) had metastatic disease at initial diagnosis, with the lung being the most common site of metastasis. Tumor thrombus was present in 8 patients, and 4 experienced local recurrence after nephrectomy. In 2023, Tang et al. (21) analyzed clinical data from 23 CDC patients. Ten patients were in stage III, and 9 were in stage IV, with over 73.9% of patients having metastatic disease at diagnosis. Lymph node metastasis occurred in 12 patients, and distant metastasis in 7, with bone metastasis being the most frequent in this study. Chen et al. (19) collected data from 74 CDC patients across two centers between 2001 and 2020. Metastasis was observed in 32 patients (43%), comprising 18 cases (24.3%) of distant metastasis and a 32.4% rate of lymph node metastasis. Bone metastasis was the next most common at 13.5%, followed by lung metastasis at 8.1%. Tumor thrombus within veins was found in 17.6% of patients. Panunzio et al. (20) identified 399 CDC patients from the SEER database between 2004 and 2018. Among them, 148 (37.1%) had lymph node involvement, and 156 (39.1%) had distant metastasis. Studies indicate that the vast majority of CDC patients are diagnosed at pT3 or higher stages (3, 5, 6, 17), and up to 71% have metastatic disease upon completion of staging (17). Symptoms related to metastasis depend on the site of involvement, with lung and bone metastases being the most common. Other frequently reported sites include the liver, skeletal muscle, brain, adrenal glands, and pleura (23). Rare metastatic sites such as the heart (11) and oral cavity have also been reported (12). There are also cases where skin nodules were biopsied and determined to be metastatic CDC (13).

**Table 2:** Summary of CDC lymph node and distant metastasis.

No	Year	Author	People	Lymphatic metastasis	Distant metastasis (the commonest)	Reference
1	2006	Tokuda	81	19 (44.2%) (LND:43)	26 (32.1%) (Lung)	(5)
2	2007	Karakiewicz	41	20 (48.8%)	8 (19.5%) (NR)	(6)
3	2017	Sui	577	277 (48.0%)	184 (31.9%) (Lung)	(17)
4	2022	Xie	21	11 (52%)	7 (33%) (Lung)	(18)
5	2022	Chen	74	24 (32.4%)	18 (24.3%) (Bone)	(19)
6	2023	Panunzio	399	148 (37.1%)	156 (39.1%) (NR)	(20)
7	2023	Tang	23	12 (52.2%)	7 (30.4%) (Bone)	(21)

NR, No Reported; LND, Lymph node dissection.

CDC is a highly aggressive subtype of renal cell carcinoma. The most common clinical symptom is gross hematuria, followed by flank pain or an abdominal mass. Some patients may experience fatigue, weight loss, and other systemic symptoms. Pathologically, the tumor typically presents as high-grade, advanced-stage, and highly malignant (24). In this case, the patient was admitted with a one-week history of hematuria and no additional symptoms. Therefore, after further comprehensive examinations were completed during hospitalization, elective surgical treatment was scheduled.

The initial diagnosis of CDC is similar to that of common renal cell carcinomas and can be assisted preoperatively by renal ultrasound, CT, and MRI (15). On CT, collecting duct carcinoma (CDC) typically appears as an infiltrative mass originating from the renal medulla and extending into the renal sinus (often with relative preservation of the renal contour). It may contain cystic areas and calcifications, and shows weak to moderate, heterogeneous enhancement on contrast administration, as seen in the present case. However, intraoperative and postoperative pathological examination remains the gold standard for definitive diagnosis.

The International Society of Urological Pathology in 2013 and the newly revised World Health Organization classification in 2016 (25, 26) specify that the diagnostic criteria for CDC must include the following: high-grade nuclear features, a tumor located in the renal medulla, exhibiting tubular architecture, a desmoplastic stroma, and infiltrative growth. The most crucial point is the absence of any other malignant tumor components upon extensive pathological sampling, such as other subtypes of renal cell carcinoma or urothelial carcinoma. Therefore, a definitive diagnosis of CDC requires differentiation from other malignancies (27), such as high-grade urothelial carcinoma (HGUC) of the renal pelvis, renal medullary carcinoma (RMC), fumarate hydratase (FH)-deficient renal cell carcinoma, and anaplastic lymphoma kinase (ALK)-rearranged renal cell carcinoma (ALK-RCC) (**Table 3**).

**Table 3:** Differential diagnosis among diseases.

Disease	CDC	HGUC	RMC	FH-RCC	ALK-RCC
Incidence of disease	<2%	<10%	<1%	<2%	<1%
Clinical Manifestation	Middle-aged, Hematuria, Low back pain, Abdominal mass, systemic symptoms.	Elderly, Hematuria, Low back pain, Hydronephrosis, Urinary tract irritation	20–30 years, Hematuria, Low back pain, Sickle cell anemia, Systemic symptoms	30–40 years, Hematuria, History of skin/uterine smooth muscle tumors	Children/Adolescents Hematuria Low back pain Abdominal mass
Imaging characteristics	Infiltrative mass of the renal medulla, extending into the renal sinus, which may contain cystic areas and the focus of calcification	Filling defect in the renal pelvis with dilated calyces	Infiltrating mass in the center of the renal medulla with renal pelvic invasion	Cortical mass with spoke-like enhancement and cystic degeneration	Cortical solid or cystic swelling
Pathology (Morphology and IHC)	Irregular glandular tubular structure promoting fibroproliferative interstitium. 34βE12(+) PAX8(+) INI-1(+) p63(-) OCT3/4(-)	Papillary, nested/solid, infiltrative. PAX8(-) p63(+) GATA3(+) p40(+) CK7(+) CK20(+)	Reticulated/screened, mucus-like interstitium + neutrophils. INI-1 (-) OCT3/4(+) PAX8(+)	Papillary, tubular cystic, giant eosinophilic nuclei. PAX8(+) INI-1 (+) 2SC(+) FH(+/-) OCT3/4(-)	Solid/follicular/papillary + intracytoplasmic mucus vacuoles. PAX8(+) ALK (+)
Treatment	RN + ST	RNU+BCE + ST	RN + ST	RN + ST	RN + ST
Prognosis	Extremely poor.	Relatively poor	Extremely poor.	Poor	Relatively good (ALK-TKI sensitive)

CDC, Collecting Duct Carcinoma; HGUC, High-Grade Urothelial Carcinoma; RMC, Renal Medullary Carcinoma; FH-RCC, Fumarate Hydratase-deficient RCC; ALK-RCC, Anaplastic Lymphoma Kinase -rearranged RCC. RCC, Renal Cell Carcinoma. RN, Radical Nephrectomy; ST, Systemic Therapy; RNU, Radical Nephroureterectomy; BCE, Bladder Cuff Excision; IHC, Immunohistochemistry.

The primary site of CDC involvement is the renal medulla, with possible extension into the cortex when the tumor reaches a larger size (28, 29). Its gross features include a gray-white or tan-white cut surface, firm consistency, ill-defined borders, and the presence of necrosis and hemorrhage. Invasion into the perirenal fat, renal hilum, and/or renal veins is commonly observed (30).

When histomorphology raises suspicion for CDC, immunohistochemistry (IHC) becomes particularly important. While IHC cannot directly diagnose CDC, it provides crucial evidence for excluding other entities and ultimately confirming the diagnosis. CDC exhibits a broad immunophenotypic spectrum and lacks specific markers. Although the expression patterns of high molecular weight cytokeratins such as 34βE12, CK19, and CK7 have been used to identify CDC (31, 32), their positivity rates are variable and their diagnostic utility is limited. Therefore, it is recommended to employ a diagnostic antibody panel including PAX8, OCT3/4, SMARCB1 (INI-1), p63/p40, GATA3, FH/2SC, and ALK for auxiliary diagnosis. This panel aids in excluding other tumor entities in the differential diagnosis. The flexible application of this combination can facilitate the exclusionary diagnosis of other subtypes of renal cell carcinoma and urothelial carcinoma. Positive PAX8 expression combined with negative p63 is helpful in distinguishing CDC from urothelial carcinoma (33). Loss of SMARCB1 (INI-1) expression with negative OCT3/4 aids in excluding renal medullary carcinoma and undifferentiated carcinoma (34, 35). A profile of PAX-8 negative, p63 positive, and GATA3 positive typically suggests high-grade urothelial carcinoma (36). For FH-deficient renal cell carcinoma, the characteristic immunohistochemical features include PAX-8 positive, SMARCB1 (INI-1) positive, OCT3/4 negative, along with an FH(-)/2SC(+) or FH(+/-)/2SC(+) pattern; these findings strongly support the diagnosis (37).

Although CDC is classified as a subtype of renal cell carcinoma, its clinical presentation, tumor biology, imaging characteristics, and prognosis are distinctly different from other types of renal cancer. The extreme rarity of CDC has resulted in limited molecular research, which in turn contributes to a lack of established treatment strategies for metastatic CDC (mCDC), ultimately leading to poor prognoses for these patients. The primary treatment for CDC remains radical surgery (38, 39), but this must be carefully considered in the context of the patient’s ability to tolerate the procedure and the presence or absence of distant metastasis.

The surgical decision-making in this case adhered to an individualized principle centered on safely achieving effective cytoreduction. The preoperative clinical diagnosis was metastatic (Stage IV) collecting duct carcinoma. Given that adrenal biopsy carried the risk of injuring adjacent major blood vessels, the surgical objective was defined as a cytoreductive nephrectomy. Intraoperative exploration revealed that the enlarged lymph nodes in the renal hilum and retroperitoneal area were densely adherent to the renal vascular pedicle (particularly the renal vein), obscuring clear anatomical planes. Forcing an extended lymph node dissection or ipsilateral adrenalectomy under these circumstances would have significantly increased the risk of damaging critical vasculature and causing iatrogenic tumor dissemination. Therefore, the surgical team prudently decided to focus on the safe and complete resection of the primary renal tumor, without performing radical resection of the high-risk areas. This decision-making process highlights the importance of intraoperative real-time risk-benefit assessment in adjusting the surgical scope for patients with advanced renal cell carcinoma in the context of radiologically suspected metastases.

This case presents an intriguing clinical paradox: preoperative contrast-enhanced CT highly suspected ipsilateral adrenal and regional lymph node metastases, leading to the clinical decision to perform a cytoreductive nephrectomy for stage IV kidney cancer. However, the patient did not receive any adjuvant systemic therapy postoperatively. Follow-up imaging at the 13-month mark (as of manuscript submission) revealed no evidence of recurrence or metastasis. This outcome prompts a re-evaluation of the preoperative imaging assessment. The ipsilateral adrenal lesion seen on CT was highly likely a benign condition, such as a lipid-poor adrenal adenoma, as both this entity and metastatic lesions can exhibit heterogeneous enhancement on CT. This case strongly underscores that for imaging-indicated, particularly solitary and suspicious metastatic foci such as in the adrenal gland, every effort should be made to obtain pathological confirmation before making major therapeutic decisions that could alter treatment goals (e.g., shifting from curative-intent to cytoreductive surgery). The benefits and risks of percutaneous biopsy in this context, as well as its value in avoiding “over-staging” and “over-treatment,” warrant discussion.

Preoperative imaging strongly suggested stage IV collecting duct carcinoma (cT3aN1M1), and a cytoreductive nephrectomy was performed accordingly. However, the patient achieved long-term disease-free survival for 13 months postoperatively without any adjuvant therapy. This discrepancy could be explained by the preoperative CT potentially misinterpreting a benign adrenal lesion (such as a lipid-poor adenoma) as metastasis, leading to “clinical over-staging.” Although the initial decision was based on the highly aggressive nature of collecting duct carcinoma and the imaging evidence available at the time, aligning with the principle of safety-first, this case serves as a profound warning. For solitary suspicious metastatic lesions—especially in the adrenal gland—that determine therapeutic goals, every effort should be made to achieve pathological confirmation through multidisciplinary discussion and biopsy to enable accurate staging and avoid overtreatment. Simultaneously, it vividly illustrates how close follow-up can ultimately revise diagnosis and staging, and demonstrates that even with suspicion of minimal metastatic disease, some patients may achieve excellent outcomes following aggressive local management of the primary tumor. Its value lies not only in being a rare record of collecting duct carcinoma with a favorable prognosis but also in serving as a compelling example that prompts clinicians to reflect on staging pitfalls in renal cancer and to weigh diagnostic approaches against therapeutic decisions.

While most CDC patients undergo surgical treatment, the aggressive nature of the disease often leads to rapid postoperative recurrence and metastasis. There is a lack of consensus regarding the treatment strategy for metastatic CDC (mCDC). Based on shared biological characteristics with urothelial carcinoma, the National Comprehensive Cancer Network (NCCN) guidelines for kidney cancer recommend platinum-based chemotherapy regimens used for urothelial carcinoma as the standard first-line treatment for mCDC (40). However, according to previous studies (**Table 4**), Tokuda et al. (5) retrospectively analyzed 49 CDC patients who received various adjuvant therapies (chemotherapy in 17, radiotherapy in 3, and immunotherapy in 34) and found that only one patient (2.0%) with lung metastasis exhibited a partial response (PR) to a gemcitabine plus carboplatin regimen. Oudard et al. (41) conducted a multicenter phase II prospective study evaluating gemcitabine plus platinum-based regimens as first-line therapy in 23 patients with metastatic collecting duct carcinoma. The results showed a complete response (CR) rate of only 4.3%, a partial response (PR) rate of 21.7%, a median progression-free survival (PFS) of 7.1 months, and a median overall survival (OS) of 10.5 months. In contrast, Watanabe et al. (44) reported a case of CDC with multiple lymph node metastases achieving a complete response (CR) following treatment with nivolumab combined with ipilimumab after partial nephrectomy. Sheng et al. (43) conducted a prospective, multicenter phase II study involving 26 treatment-naïve metastatic CDC patients, evaluating a triple regimen of gemcitabine, cisplatin, and sorafenib. This regimen demonstrated significant efficacy: the 6-month progression-free survival rate reached 65.0%, with a median PFS of 8.8 months (95% CI: 6.7-10.9) and a median OS of 12.5 months (95% CI: 9.6-15.4). The objective response rate (ORR) increased to 30.8%, and the disease control rate (DCR) was as high as 84.6%, with treatment-related adverse events mostly being mild to moderate. Zeng et al. (14) utilized whole-exome sequencing and transcriptome sequencing to identify patient-specific neoantigens. They subsequently synthesized neoantigen long-peptide vaccines and neoantigen-reactive T cells (NRTs), evaluating the immune response via ELISPOT assay. Personalized neoantigen-based immunotherapy elicited significant immune responses and delayed disease progression in specific cases, suggesting its potential value for mCDC. Bratslavsky et al. (48), by comparing the genomic profiles of different renal cancer types, revealed that mCDC is genetically distinct, with limited targeted effective treatments and inadequate responses to conventional immunotherapy. Pecuchet et al. (42) found that the combination of bevacizumab, gemcitabine, and platinum agents could prolong both PFS and OS in mCDC patients. Procopio et al. (45) prospectively evaluated the efficacy of cabozantinib in 23 mCDC patients, with encouraging results: one patient achieved complete response, three had stable disease, and seven showed partial response, yielding an ORR of 35% (95% CI: 16%-57%). The median PFS was 4 months (95% CI: 3–13 months), and the median OS was 7 months (95% CI: 3–31 months). Buti et al. (46) investigated nivolumab as an option after failure of first-line cabozantinib for mRCC. Their results from four patients receiving immunotherapy showed one partial response and two with stable disease. The second-line PFS ranged from 2.8 to 19.9 months, second-line OS from 5.1 to 26.5 months, with an ORR of 25%, suggesting nivolumab could be a second-line treatment option. In 2023, Funajima et al. (47) reported a case of mCDC achieving a complete response (CR) with combined nivolumab and cabozantinib treatment.

**Table 4:** Summary of mCDC treatment and evaluation of antitumor efficacy endpoints.

No	Study design	Year of publication	Author	Patients	Chemotherapeutic regimens	Primary endpoint	Ref
1	Retrospective	2006	Tokuda	49	17 Chemotherapy 3 Radiotherapy 34 Immunotherapy	1 PR(GC)	(5)
2	Prospective	2007	Oudard	23	Gemcitabine +Platinum	4.3% CR 21.7% PR ORR 26% PFS 7.1months OS 10.5 months	(41)
3	Retrospective	2013	Pecuchet	5	Bevacizumab +Gecitabine +Platinum	ORR 60% PFS 15.1 months OS 27.8 months	(42)
4	Prospective	2018	Sheng	26	Gemcitabine +Cisplatin +Sorafenib	ORR 30.8% DCR84.6% PFS 8.8months OS12.5 months	(43)
5	Retrospective	2020	Watanabe	1	Nivolumab +Ipilimumab	CR	(44)
6	Retrospective	2020	Zeng	1	Neoantigen-based immunotherapy	SD	(14)
7	Prospective	2022	Procopio	23	Cabozantinib	1 CR 3 SD 7 PR ORR 35% PFS 4 months OS 7 months	(45)
8	Retrospective	2022	Buti	4	Nivolumab	ORR 25% PFS 2.8-19.9months OS 5.1-26.5months	(46)
9	Retrospective	2023	Funajima	1	Nivolumab +ipilimumab	CR	(47)

GC, gemcitabine and carboplatin; CR, Complete response; PR, Partial response; ORR, Objective response rate; PFS, Progression-Free Survival; OS, Overall Survival; DCR, Disease Control Rate; SD, Stable disease; Ref, Reference.

Metastatic collecting duct carcinoma (CDC) carries an extremely poor prognosis in kidney transplant recipients. Recent research has revealed that the BK polyomavirus (BKPyV) is a key oncogenic driver within the immunosuppressive microenvironment, providing a novel perspective for understanding and managing this type of tumor. The oncogenicity of BKPyV has been strongly substantiated at both the protein and genomic levels. The study by Dao et al. (49) demonstrated that in transplant-associated CDC, the BKPyV large T antigen (SV40) is specifically and persistently expressed in the nuclei of tumor cells, while being negative in non-tumor tissues, strongly suggesting its direct carcinogenic role. More groundbreaking evidence comes from Meier et al. (50), who, through whole-genome sequencing, were the first to directly identify genomic integration of BKPyV at multiple chromosomal loci (e.g., chromosomes 2, 4, 6, 9) in metastatic CDC. This finding establishes a molecular-level link between viral integration and tumorigenesis, defining post-transplant CDC as a virally driven malignant disease. This understanding has fundamentally altered clinical management strategies and created new therapeutic opportunities. For localized disease, the case presented by Dao et al. demonstrates that maintaining immunosuppression with close monitoring is a viable option to preserve the function of a vital graft (49). However, for more aggressive metastatic disease, a radical strategy of immune reconstitution should be pursued. The successful paradigm provided by Meier et al. is crucial: in a case with extensive metastases, the complete withdrawal of immunosuppression combined with IL-2 therapy successfully activated multiple host immune responses, ultimately achieving complete remission and disease-free survival for over six years (50). This proves that reversing the immunosuppressed state may offer hope for a cure in advanced patients.

In summary, we advocate for a paradigm shift in the management of post-transplant CDC: A history of BKPyV infection should be recognized as a significant risk factor, warranting regular radiographic surveillance. Treatment decisions should be precisely tailored between “conservative monitoring” and “aggressive immune reconstitution” based on the tumor stage and the functional status of the graft. Future research should focus on developing targeted or immunotherapies directed against BKPyV antigens, aiming to provide more effective and specific treatments for these patients.

The aggressive clinical course of mCDC highlights the limitations of current treatment options. When discussing the highly challenging disease of metastatic CDC, its biological similarities to urothelial carcinoma provide a rationale for exploring novel therapeutic targets. Preliminary results from the CICERONE study, recently presented at the 2025 ASCO Annual Meeting, offer key evidence in this direction. This study is the first to systematically evaluate the expression of two important antibody-drug conjugate (ADC) targets—Nectin-4 and TROP-2—in 59 metastatic CDC samples. It found an expression rate of up to 98% for TROP-2, while Nectin-4 was expressed in 41% of cases (51). This discovery not only supports the hypothesis of molecular similarity between CDC and urothelial carcinoma but, more importantly, reveals for the first time that ADC-based therapies may represent a highly promising new direction for treating metastatic CDC. Based on this robust target expression data and the successful experience of pembrolizumab combined with ADCs in urothelial carcinoma (e.g., the EV-302 trial), researchers have proposed applying similar combination regimens to CDC, with plans for validation in the phase II RePRINT trial.

The studies discussed above collectively indicate that the efficacy of currently recommended chemotherapy, targeted therapy, and immunotherapy remains highly controversial. Large-scale genomic sequencing is still required to further elucidate the mechanisms underlying the development and progression of collecting duct carcinoma, with the goal of identifying more precise molecular targets for the effective treatment of mCDC.

## Conclusion

The management of collecting duct carcinoma (CDC) poses significant challenges. The discrepancy observed in this case between “radiologically suspected metastasis” and “long-term disease-free survival postoperatively” underscores the critical importance of pathological confirmation for suspected metastatic lesions to avoid overtreatment. It also suggests that aggressive local control may benefit a subset of patients. Current research is reshaping the therapeutic paradigm for CDC: in transplant-associated cases, the oncogenic mechanism of BK polyomavirus provides a new target for exploring immunotherapies (such as immunosuppression withdrawal); while in sporadic cases, the high expression of Nectin-4/TROP-2 establishes a foundation for antibody-drug conjugate (ADC) therapy. Moving forward, the management of CDC requires a dual focus on clinical precision and scientific innovation: in practice, stratified management should be achieved through multidisciplinary team (MDT) collaboration; in research, novel therapies targeting viral antigens, ADC targets, and others need to be validated. This integrated approach will propel this refractory tumor into the era of precision medicine.

## Data Availability

The original contributions presented in the study are included in the article/supplementary material. Further inquiries can be directed to the corresponding authors.
